# Identifying nucleotide-binding leucine-rich repeat receptor and pathogen effector pairing using transfer-learning and bilinear attention network

**DOI:** 10.1093/bioinformatics/btae581

**Published:** 2024-09-27

**Authors:** Baixue Qiao, Shuda Wang, Mingjun Hou, Haodi Chen, Zhengwenyang Zhou, Xueying Xie, Shaozi Pang, Chunxue Yang, Fenglong Yang, Quan Zou, Shanwen Sun

**Affiliations:** Key Laboratory of Saline-Alkali Vegetation Ecology Restoration, Ministry of Education (Northeast Forestry University), Harbin 150001, China; State Key Laboratory of Tree Genetics and Breeding, Northeast Forestry University, Harbin 150001, China; Key Laboratory of Saline-Alkali Vegetation Ecology Restoration, Ministry of Education (Northeast Forestry University), Harbin 150001, China; State Key Laboratory of Tree Genetics and Breeding, Northeast Forestry University, Harbin 150001, China; Key Laboratory of Saline-Alkali Vegetation Ecology Restoration, Ministry of Education (Northeast Forestry University), Harbin 150001, China; Key Laboratory of Saline-Alkali Vegetation Ecology Restoration, Ministry of Education (Northeast Forestry University), Harbin 150001, China; Key Laboratory of Saline-Alkali Vegetation Ecology Restoration, Ministry of Education (Northeast Forestry University), Harbin 150001, China; Key Laboratory of Saline-Alkali Vegetation Ecology Restoration, Ministry of Education (Northeast Forestry University), Harbin 150001, China; Key Laboratory of Saline-Alkali Vegetation Ecology Restoration, Ministry of Education (Northeast Forestry University), Harbin 150001, China; College of Landscape Architecture, Northeast Forestry University, Harbin 150001, China; Department of Bioinformatics, Fujian Key Laboratory of Medical Bioinformatics, School of Medical Technology and Engineering, Fujian Medical University, Fuzhou 350122, China; Key Laboratory of Ministry of Education for Gastrointestinal Cancer, School of Basic Medical Sciences, Fujian Medical University, Fuzhou 350122, China; Institute of Fundamental and Frontier Sciences, University of Electronic Science and Technology of China, Chengdu 611731, China; Key Laboratory of Saline-Alkali Vegetation Ecology Restoration, Ministry of Education (Northeast Forestry University), Harbin 150001, China; State Key Laboratory of Tree Genetics and Breeding, Northeast Forestry University, Harbin 150001, China

## Abstract

**Motivation:**

Nucleotide-binding leucine-rich repeat (NLR) family is a class of immune receptors capable of detecting and defending against pathogen invasion. They have been widely used in crop breeding. Notably, the correspondence between NLRs and effectors (CNE) determines the applicability and effectiveness of NLRs. Unfortunately, CNE data is very scarce. In fact, we’ve found a substantial 91 291 NLRs confirmed via wet experiments and bioinformatics methods but only 387 CNEs are recognized, which greatly restricts the potential application of NLRs.

**Results:**

We propose a deep learning algorithm called ProNEP to identify NLR-effector pairs in a high-throughput manner. Specifically, we conceptualized the CNE prediction task as a protein–protein interaction (PPI) prediction task. Then, ProNEP predicts the interaction between NLRs and effectors by combining the transfer learning with a bilinear attention network. ProNEP achieves superior performance against state-of-the-art models designed for PPI predictions. Based on ProNEP, we conduct extensive identification of potential CNEs for 91 291 NLRs. With the rapid accumulation of genomic data, we expect that this tool will be widely used to predict CNEs in new species, advancing biology, immunology, and breeding.

**Availability and implementation:**

The ProNEP is available at http://nerrd.cn/#/prediction. The project code is available at https://github.com/QiaoYJYJ/ProNEP.

## 1 Introduction

Pathogens are the main biological stresses faced by plants (Singh *et al.* 2023). They cause great economic losses (Trumbore *et al.* 2015) and pose a serious threat to global food security. Some plants have evolved sophisticated immune systems to sense pathogens and activate the immune responses to fight them. One of the key components of the immune system is the nucleotide-binding leucine-rich repeat (NLR) receptors family (Liu *et al.* 2023). It is activated by virulence proteins (i.e. effectors; Contreras *et al.* 2023) secreted by pathogens, triggering hypersensitivity and cell death (Wan *et al.* 2019) to fight pathogen invasion. NLR has attracted increasing attention recently. A large number of NLRs have been found (Jones *et al.* 2016, Liu *et al.* 2021, Calle García *et al.* 2022), and some NLRs have been used for breeding disease-resistant crops. For example, *Rpi-blb2* was originally identified in *Solanum bulbocastanum* as an NLR to resist the late blight disease caused by *Phytophthora infestans* (Orbegozo *et al.* 2016). The gene encoding *Rpi-blb2* is transferred to *Solanum demissum* to confer a broad-spectrum resistance to isolates of *P.infestans* (Haverkort *et al.* 2009, Wu *et al.* 2017), which can greatly reduce the costs of disease control and increase the yield of potatoes (Haverkort *et al.* 2008). To successfully apply NLRs in resistance breeding, identifying the targets (i.e. pathogen effectors) of NLRs is crucial (Białas *et al.* 2018). Wet experiment is the main and reliable method to identify the presence of NLR and effector pairs (i.e. the correspondence between NLRs and effectors; CNE), but it requires high experimental cost and a long experimental period (Dao *et al.* 2019). Therefore, CNE data is very scarce. In fact, some databases have collected NLR data but there is no CNE information (Liu *et al.* 2021, Li *et al.* 2023). Through a systematic review of the literature, we identified a total of 91 291 NLRs (Liu *et al.* 2021) that have been verified through wet experiments and bioinformatics methods. In contrast, only 387 CNEs of 259 NLRs have been experimentally confirmed. The limited number of CNEs restricts the application of NLRs in breeding practice. Therefore, accurately identifying CNEs on large-scale data becomes urgent.

Deep learning is one of the fastest-growing topics in biology (Tang *et al.* 2021, Ao *et al.* 2022, Choudhary *et al.* 2022, Li *et al.* 2023, Wang *et al.* 2023, Yan *et al.* 2023) and achieved great success in the last few years (Zeng *et al.* 2022). Especially in protein fields, deep learning has been used extensively (Chen *et al.* 2023), such as protein structure prediction models (e.g. AlphaFold2; Jumper *et al.* 2019), drug-target interaction (DTI) prediction models (e.g. GraphDTA; Nguyen *et al.* 2021), and protein–protein interaction (PPI) prediction models (e.g. Topsy-Turvy; Singh *et al.* 2022). One big advantage of deep learning in solving protein problems is that it can learn from large volumes of protein data to embed them into a complex latent feature space (Lin *et al.* 2023). In addition, large protein language models together with attention mechanisms (Bahdanau *et al.* 2014) can learn interactions among sequence elements (Kaplan *et al.* 2020, Li *et al.* 2021, Hoffmann *et al.* 2022, Nijkamp *et al.* 2022, Song *et al.* 2022, Zhang *et al.* 2022). Deep learning is also capable of processing and integrating protein information from various data sources (Kulmanov *et al.* 2018), such as sequences (Chowdhury *et al.* 2022, Jin *et al.* 2022, Wang *et al.* 2023), structures (Berman *et al.* 2000), interaction networks (Szklarczyk *et al.* 2019), and even domain information (Mistry *et al.* 2021). Through this integration, deep learning models can capture the complex relationships and interdependencies between different modalities, thereby enhancing the accuracy and robustness of automatic predictions (Sapoval *et al.* 2022, Ao *et al.* 2023). However, to our best knowledge, there are currently no automatic tools to identify CNEs.

In this study, we conceptualized CNE identification as the problem of PPI prediction based on the fact that NLR and effector interact directly or indirectly through guard proteins or bait proteins in the process of immune response. Although we may benefit from the latest deep learning methods in PPI predictions, two challenges remain in our work. The first challenge is how to build a well-performing deep learning model based on a limited dataset (387 CNEs, verified by traditional wet experiments; see above). The sparsity of the training data remains a fundamental challenge for deep learning approaches (Lake *et al.* 2017). The second challenge is how to learn the pairing of NLR and effector. CNE is essentially determined by the interactions between residues in NLR and effector. The ability to simulate complex interactions between two inputs is critical to CNE identifications. In previous PPI studies, the focus has been on capturing position-wise interactions between residues by applying element-wise operations (e.g. sum and product) over protein features (Sledzieski *et al.* 2021). Learning all pairwise interactions between residues still requires further exploration.

To address these challenges, we presented a computational method for CNE identification called ProNEP based on transfer learning and the bilinear attention network (Kim *et al.* 2018, Yu *et al.* 2018). Transfer learning based on pre-trained models has proven successful in both natural language processing (Peters *et al.* 2018, Devlin *et al.* 2019) and pattern recognition domains (Shin *et al.* 2016). They can make use of the large data from related tasks to improve the performance of the target task with limited data. We first transferred protein knowledge from a pre-trained model from Bepler and Berger [a semi-supervised bidirectional long short-term memory (Bi-LSTMs) neural network] to embed NLRs and effectors to generate sequence features (X_N_ and X_E_). X_N_ and X_E_ capture the local and global information of the structure, function, and evolution of NLRs and effectors sequences, respectively. Then, the dimensions of X_N_ and X_E_ are reduced using the Convolutional Neural Network (CNN) module to obtain SN and SE which capture a series of local residue patterns by expanding the receptive field through a chain of convolutional layers. Next, S_N_ and S_E_ are fed into a pairwise interaction module that consists of a bilinear attention network to output a joint representation that learns detailed interactions between all residues of NLRs and effectors in a multiplicative way. Finally, we used an Multilayer Perceptron (MLP) to decode the joint representation into the CNE prediction. The evaluation on the independent test dataset shows that ProNEP demonstrates strong performance, with an Area Under the Receiver Operating Characteristic Curve (AUROC) of 0.966 and an Area Under the Precision-Recall Curve (AUPRC) of 0.747. The evaluation on two unseen datasets where 10% NLRs and effectors were deliberately excluded from the training and validation sets, respectively, shows the robust generalization of ProNEP, with both AUROC exceeding 0.90 and both AUPRC over 0.69. Moreover, ProNEP accurately predicted all CNEs in nine newly published datasets. These results suggest that ProNEP can rapidly and accurately identify potential CNEs on a large scale, thereby advancing species conservation and agricultural production. In summary, the main contributions of ProNEP are: (i) it is the first, to our knowledge, CNE prediction model that can work in a high-throughput way based solely on sequence data; (ii) it introduces a bilinear attention mechanism to capture all the residue interactions between NLRs and effectors.

## 2 Methods

### 2.1 Pre-trained module

The ProNEP model takes as inputs an NLR sequence of length *m* and an effector sequence of length *n*. We generated feature embeddings X_N_ ∈ R^*m*^ ^× 6165^ and X_E_ ∈ R^*n*^ ^× 6165^ by embedding the NLRs and the effectors, respectively, using the pre-trained model from Bepler and Berger (2021). This pre-trained model is a supervised Bi-LSTM neural network. The bidirectional architecture enables the pre-trained model to effectively consider contextual dependencies in both the forward and backward directions, extracting more comprehensive protein features. By leveraging transfer learning based on the pre-trained model, we transferred the generic features and knowledge learned from large-scale protein sequence data to the CNE prediction problem. Overall, X_N_ and X_E_ captured the local and global features of structure, function, and evolution of NLRs and effectors, respectively.

### 2.2 CNN module

The CNN module consists of four consecutive 1D-convolutional layers. The initial convolutional layer is employed to capture local residue patterns using a kernel size of 3. Subsequently, the next three layers continue to expand the receptive field, allowing the CNN module to extract longer or larger local residue patterns from the protein feature matrix X_N_ and X_E_ while achieving dimensionality reduction. In each convolutional layer, a convolution operation is performed on the input matrix, followed by a non-linear transformation through the Rectified Linear Unit (ReLU) activation function. The CNN module is written as:


(1) $$S_{N}^{\left(l+1\right)}=\mathrm{ReLU}\left(\mathrm{CNN}\left(W_{N}^{\left(l\right)},b_{N}^{\left(l\right)},S_{N}^{\left(l\right)}\right)\right),$$

(2) $$S_{E}^{\left(l+1\right)}=\mathrm{ReLU}\left(\mathrm{CNN}\left(W_{E}^{\left(l\right)},b_{E}^{\left(l\right)},S_{E}^{\left(l\right)}\right)\right),$$

where ***S_N_*** ∈ R^*m*^ ^×^ ^*d*^^0^ and ***S_E_*** ∈ R^*n*^ ^×^ ^*d*^^0^ are the final feature representations for NLRs and the effectors, $W_{N}^{\left(l\right)}\;$and $W_{E}^{\left(l\right)}\;$denote the learnable weight matrices, $b_{N}^{\left(l\right)}\;$and $b_{E}^{\left(l\right)}$ denote the bias vectors in the *l*th CNN layer. For any given layer *l*, $S_{N}^{\left(l\right)}\;\mathrm{and}\;S_{E}^{\left(l\right)}$ denote the *l*th hidden protein representation. Initially, $S_{N}^{\left(0\right)}\;$is X_N_ and $S_{E}^{\left(0\right)}$ is X_E_.

### 2.3 Interaction learning module

Here, we introduced the multi-head bilinear attention network (Kim *et al.* 2018) to obtain all pairwise interactions between NLR and effector proteins. This bilinear approach effectively reduces the input dimensionality, thereby decreasing computational demands, while simultaneously enhancing the granularity of residue interaction information.

Upon applying a CNN model for dimensionality reduction, we obtained the feature vectors *S_N_* ∈ R^*m*^ ^×^ ^*d*^^0^ and *S_E_* ∈ R^*n*^ ^×^ ^*d*^^0^ for the respective input sequences. A single-head pairwise interaction ***M*** ∈ R^*d*0 × *d*0^ can be obtained by the Hadamard calculation of the bilinear interaction map:


(3) $$M=\mathrm{softmax}\left(\left(\left(1\cdot P^{T}\right)○\left({S_{N}}^{T}U\right)\right)\left(V^{T}S_{E}\right)\right),$$

where **U** ∈ R^*m*^^×^^*k*^ and **V** ∈ R^*m*^^×^^*k*^ are learnable weight matrix of NLR and effector features, respectively. P ∈ R^*k*^ is a learnable weight vector, 1 ∈ R^*d*^^0^ represents a fixed all-ones vector, and ○ denotes Hadamard calculation. Specifically, each element in Equation (3) can be represented by:


(4) $$M_{i,j}=P^{T}\left(\left(U^{T}{(S}_{N_{i}})\right)○\left(V^{T}{(S}_{E_{j}})\right)\right),$$

where $S_{N_{i}}$ is the *i*th column of $S_{N}\;$and $S_{E_{j}}\;$is the *j*th column of $S_{E}$, respectively, denoting the *i*th and *j*th residue representations of the NLRs and effectors.

We obtained the final joint representation **f_*h*_** through bilinear pooling layers over the interaction maps ***M***:


(5) $${f_{h}}_{k}=({{S_{N}}^{T}U_{h})}_{k}^{T}\cdot M\cdot ({{S_{E}}^{T}V_{h})}_{k},$$

(6) $${f_{h}}_{k}=\sum_{i}^{d_{0}}\sum_{j}^{d_{0}}M_{i,j}({S_{N_{i}})}^{T}\left(\left(U_{h_{k}}\right)\left(V_{h_{k}}{)}^{T}\right){(S}_{E_{j}}\right),$$

where *h* is for heads and ${f_{h}}_{k}$ denotes the *k*th element of the intermediate representation in the *h*th head.

Last, we added a sum pooling to the joint representation vectors to obtain a compact feature map:


(7) $$f=\mathrm{SumPooling}\left(f_{h},\;s\right),$$

where the SumPool(·) function is a one-dimensional and non-overlapped sum pooling operation with stride s. It reduces the dimensionality of $f_{h}\;$∈ R^*k*^ to f ∈ R^*k*^^/^^*s*^. To get the prediction probability, the joint representation **f** is fed into the decoder, which is an MLP followed by a sigmoid function:


(8) $$p=\mathrm{Sigmoid}\left(\mathrm{Wf}+b\right),$$

Finally, we jointly optimized all learnable parameters by backpropagation. The training objective is to minimize the cross-entropy loss as follows:


(9) $$L=-\frac{1}{N}\sum_{i}[y_{i}\cdot \log\left(p_{i}\right)+\left(1-y_{i}\right)\cdot \log\left(1-p_{i})\right],$$

where $y_{i}$ is the ground-truth label of the *i*th NLR-effector pair and $p_{i}$ is the output probability of the model.

### 2.4 Experimental settings

We implemented ProNEP in PyTorch 2.1.0. $L_{N}$ and $L_{E}$ were set to the maximum sequence length of NLRs and effectors, respectively, with padding. The pre-trained module generated a 6165-dimensional feature vector representation. CNN reduced the feature dimension with the number of filters [128, 128, 128, 128] and corresponding kernel sizes [3, 5, 7, 4]. In the bilinear attention module, we employed two attention heads. The MLP was configured with an input layer of size 256, a single hidden layer of size 512, and an output vector of size 2. During training, a batch size of 16 is utilized, and the Adam optimizer with a learning rate of 5 × 10^−5^ is applied. The models are trained for 100 epochs to ensure convergence. We selected the model with the best AUROC performance on the validation dataset and then evaluated the final performance on the test dataset.

We conducted a comparative analysis involving six models: Support Vector Machine (SVM), Random Forest (RF), D-SCRIPT, Topsy-Turvy, PIPR, and Alphafold3. To construct SVM and RF models, we utilized pretrained models to obtain feature representations of the sequence data. However, the model performance was poor, with AUROC and AUPRC scores reaching only 0.6771 and 0.382 at best, respectively. Alternatively, we employed Profeat to extract 2001-dimensional routine sequence features, capturing protein structural and physicochemical properties (Li *et al.* 2006), to build up SVM and RF models. D-SCRIPT leveraged sequence dissimilarity calculations to generate contact maps and employed a CNN-based interaction module to learn the interactions among sequence residues. Topsy-Turvy represents an enhancement to the D-SCRIPT model integrating both bottom-up (inferring properties from the characteristics of the individual protein sequences) and top-down (inferring properties from the pattern of already known PPIs in the species of interest) approaches. PIPR incorporates a deep residual recurrent convolutional neural network in the Siamese architecture. AlphaFold3 is the latest tool capable of efficiently and accurately predicting protein complex structures. When training these models, we used a threshold that maximized the F1 score. SVM with Radial Basis Function (RBF) kernel and RF are trained with the default parameters in scikit-learn. For the other four models, we followed the hyperparameter values used in the original literature. To address the issue of imbalanced training data, we incorporated a weighted-random-sampler in all the mentioned models.

## 3 Results

### 3.1 Datasets

We have collected data on NLR-effector pairs that have been validated through wet experiments from the literature and created the database NERRD. The database contains information including plant species, NLR name, NLR type, NLR protein sequences, effector name, pathogen, and effector protein sequences. To develop ProNEP, we used this database to create a dataset. In the positive samples, there are 387 CNEs validated by wet experiments, including 259 NLRs and 111 effectors. We randomly paired the NLR and effector sequences from the positive samples to generate negative samples (Hashemifar *et al.* 2018) based on a positive-negative ratio of 1:10. The dataset was divided into three parts: 80% for the training dataset, 10% for the validation dataset, and 10% for the independent test dataset, with no overlap among them (Supplementary Table S1). We conducted 10 repeated experiments, each was trained with different negative samples and tested with the same independent test set (Supplementary Table S2). To assess ProNEP’s generalization capabilities on new data, we created two new datasets: unseen-NLR and unseen-effector. For the unseen-NLR dataset, we selected 10% of NLRs from the full set and chose interaction data including these proteins for the test set, ensuring that no NLRs from this set were present in the training and validation sets (Supplementary Table S3). This dataset mimics real-world applications where users predict potential CNEs by inputting NLR data not included in our dataset. The same approach was employed to generate the unseen-effector dataset (Supplementary Table S4), which simulates a scenario where researchers predict potential CNEs by inputting their own effector data. Additionally, we tested two other pre-trained models: ProtTrans (Elnaggar *et al.* 2022) and ESM-1b (Rao *et al.* 2020). We found that ProtTrans and the Bepler and Berger model performed comparably, while ESM-1b showed weaker performance (Supplementary Table S5). We speculate that this may be due to the model’s limitations regarding sequence length. We chose the Bepler and Berger model to construct ProNEP due to its small size (Supplementary Table S5) and the requirement of online server implementation.

### 3.2 ProNEP framework

**Figure 1.**
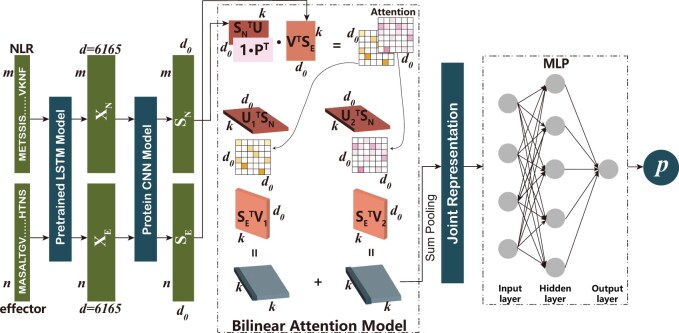
Overview of the fundamental framework of ProNEP. We first used the model proposed by Bepler and Berger as a pre-trained module to generate X_N_ ∈ R^*m*^^×6165^ and X_E_ ∈ R^*n*^^×6165^, which contain local and global information on the structure, function, and evolution of proteins. Then, X_N_ and X_E_ were transformed to S_N_ ∈ R^*m*^^×^^*d*^^0^ and S_E_ ∈ R^*n*^^×^^*d*^^0^ through the CNN module. The bilinear attention module was used to capture local interactions between S_N_ and S_E_. In this module, two attention maps were first computed with S_N_ and S_E_. Then, through the bilinear attention network, the joint representations **f_1_** and **f_2_** were calculated and summed to obtain a compact feature map **f**. Finally, an MLP was used as a classifier to output the prediction probability ***p*** ∈ [0,1] based on the final joint representation **f**

In the CNE prediction, we aim to develop a function, denoted as F, which can predict the likelihood of the interaction between a given NLR-effector pair. ProNEP takes a pair of protein sequences, denoted as $L_{N}$ and $L_{E}$, with respective lengths of *m* and *n* as input and produces an interaction probability, *p* ∈ [0, 1], as its output. It consists of four main components: a pre-trained module for extracting protein sequence features, a CNN dimension reduction module, a bilinear attention module capturing all protein residue interactions, and an MLP to output prediction probabilities, as shown in Fig. 1. We employed the pre-trained module to generate feature embeddings X_N_ ∈ R^*m*^^×6165^ and X_E_ ∈ R^*n*^^×6165^ for $L_{N}$ and $L_{E}$, capturing both local and global aspects of the protein structure, function, and evolution across various dimensions (Bepler and Berger 2021). Subsequently, the CNN module is utilized to reduce the dimensionality of the features produced by the pre-trained module, resulting in the final feature representations S_N_ ∈ R^*m*^ ^×^ ^*d*^^0^ and S_E_ ∈ R^*n*^^×^^*d*^^0^ for NLRs and the effectors, respectively. Thereafter, we deployed a bilinear attention network module to generate the joint feature representations of S_N_ and S_E_ to learn the multiplicative interactions between all residues of both proteins. Finally, the interaction probability *p* ∈ [0, 1] is predicted through an MLP. It represents the likelihood of an interaction between a given pair of NLR and effector.

### 3.3 Evaluation of ProNEP and other algorithms

**Figure 2.**
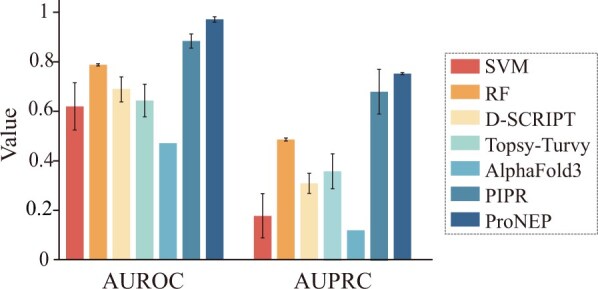
AUPRC and AUROC of SVM, RF, D-SCRIPT, Topsy-Turvy, Alphafold3, PIPR, and ProNEP on the independent test dataset

**Figure 3.**
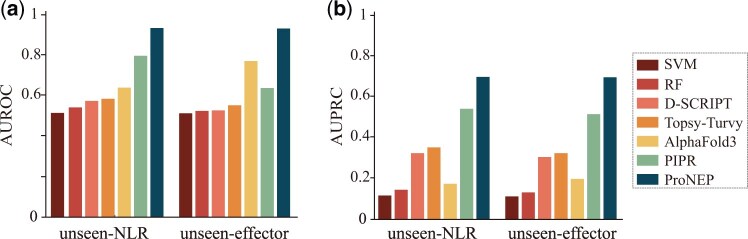
AUPRC and AUROC of SVM, RF, D-SCRIPT, Topsy-Turvy, Alphafold3, PIPR, and ProNEP on two unseen datasets

In this study, we compared ProNEP with other models, including SVM (Manganaro *et al.* 2023, Zhu *et al.* 2023, Zou *et al.* 2023), RF, D-SCRIPT (Sledzieski *et al.* 2021), Topsy-Turvy (Singh *et al.* 2022), PIPR (Chen *et al.* 2019), and Alphafold3 (Abramson *et al.* 2024). Since our training data is significantly imbalanced, with negative samples outnumbering positive samples, we used metrics, such as AUROC and AUPRC to evaluate the model performance. AUROC and AUPRC do not depend on a specific cutoff for their calculation. We chose the model with the highest AUROC performance on the validation dataset as the best-performing model, and then tested it on the independent test dataset. ProNEP achieved the highest AUROC and AUPRC, reaching 0.9685 and 0.947, respectively (Fig. 2). The second best is PIPR, with AUROC and AUPRC reaching 0.879 and 0.678 (Fig. 2). On the contrast, the performance of SVM is poor (Table 1 and Fig. 2). Although D-SCRIPT and Topsy-Turvy adopt the same feature engineering strategy as ProNEP, they do not perform as well as ProNEP (Table 1 and Fig. 2). Additionally, we tested the performance of SVM, RF, D-SCRIPT, Topsy-Turvy, Alphafold3, PIPR, and ProNEP on the unseen datasets (unseen-NLR and unseen-effector). ProNEP performs best on these two datasets, with AUROC and AUPRC scores reaching 0.9292 and 0.7134 on the unseen-NLR dataset and 0.9076 and 0.6973 on the unseen-effector dataset (Fig. 3).

**Table 1.** Performance comparison of SVM, RF, D-SCRIPT, Topsy-Turvy, Alphafold3, PIPR, and ProNEP on the independent test dataset.^a^

Model	Accuracy	Precision	Sensitivity	F1-score	AUROC	AUPRC
SVM	0.781 ± 0.024	0.157 ± 0.028	0.323 ± 0.037	0.277 ± 0.168	0.617 ± 0.065	0.176 ± 0.057
RF	0.806 ± 0.075	0.749 ± 0.086	0.610 ± 0.122	0.716 ± 0.121	0.783 ± 0.127	0.483 ± 0.117
D-SCRIPT	0.609 ± 0.073	0.324 ± 0.233	0.719 ± 0.252	0.335 ± 0.056	0.686 ± 0.065	0.306 ± 0.093
Topsy-Turvy	0.672 ± 0.117	0.225 ± 0.128	0.546 ± 0.329	0.221 ± 0.046	0.64 ± 0.046	0.359 ± 0.117
Alphafold3	0.185	0.0952	0.9189	0.1726	0.5688	0.1185
PIPR	0.912 ± 0.008	0.569 ± 0.032	0.594 ± 0.026	0.581 ± 0.026	0.879 ± 0.016	0.678 ± 0.004
ProNEP	0.914 ± 0.028	0.945 ± 0.035	0.915 ± 0.036	0.928 ± 0.022	0.966 ± 0.012	0.747 ± 0.054

a ProNEP was trained 10 times with 10 different negative samples, while the other models, excluding Alphafold3, were trained 6 times. All models, except Alphafold3, used a threshold that maximized the F1 score.

### 3.4 Ablation study

To validate the effectiveness of the pre-trained module and the bilinear attention module in predicting CNE interactions, we conducted ablation experiments. We compared the complete model with three different variants of ProNEP: No pre-trained module, unilateral attention, and linear concatenation. These three variants differ in the computation of the joint representation of NLR and effector. In the first variant, we removed the pre-trained model and used word embedding to learn protein representations. The results show that, compared with the model without the pre-training module, ProNEP’s AUROC and AUPRC increased by 39.22% and 41.33% (Table 2), respectively. This indicated that the pre-trained model can capture richer feature representations and significantly improve the model performance. In the second variant, we replaced the bilinear attention with a unilateral attention, forming two models. The unilateral attention employs the neural attention mechanism (Tsubaki *et al.* 2019), which captured the interaction strengths of an NLR as a whole with subsequences of an effector (unilateral EFF attention; Table 2) and the interaction strengths of an effector as a whole with subsequences of an NLR (unilateral NLR attention; Table 2). The AUROC of the two models were 81.95% and 82.89%, and the AUPRC were 56.21% and 50.39%, respectively. Finally, we replaced the bilinear attention with a linear concatenation. That is, after the CNN module, we simply concatenated the vector representations of NLR and effector. This variant achieves an AUROC of 55.1% and an AUPRC of 65.2% (Table 2). The results of unilateral attention and linear connection variants show that the bilinear attention mechanism captures the CNE interaction information more effectively than the two variants. Overall, the ablation study highlights the contributions of the transfer learning and the bilinear attention network to identifying CNEs.

**Table 2.** The ablation study of ProNEP on the independent test dataset.

Model	Accuracy	Precision	Sensitivity	F1-score	AUROC	AUPRC
No-pre-trained	0.835	0.8067	0.8375	0.8218	0.5683	0.3787
Unilateral NLR attention	0.8381	0.9027	0.8320	0.8659	0.8289	0.5621
Unilateral EFF attention	0.8412	0.7445	0.8512	0.7943	0.8195	0.5039
Linear concatenation	0.8375	0.89	0.8319	0.86	0.8988	0.6621
ProNEP	0.9144	0.9454	0.9145	0.9283	0.9658	0.747

### 3.5 Robustness assessment of ProNEP

In order to further evaluate the robustness of ProNEP, we selected the *Arabidopsis thaliana* and *Hyaloperonospora arabidopsidis* system with five known CNEs and *Triticum aestivum* and *Blumeria graminis* system with 20 known CNEs for testing. To construct the *A.thaliana* and *H.arabidopsidis* system dataset, we first excluded data of this system from our training and validation sets, then retrained the model. We then randomly selected 100 proteins from *A.thaliana*, mimicking the length distribution of NLR sequences, and another 100 proteins from *H.arabidopsidis*, mimicking the distribution of effectors, for testing. This process was repeated 10 times. Among these 10 datasets, 5 did not contain any positive samples (Table 3). ProNEP correctly predicted all five known CNEs and achieved a high prediction accuracy, ranging from 0.97 to 1 (Table 3). Additionally, we collected nine newly published CNE data for testing (Table 4), and ProNEP accurately predicted all of them. Overall, the results indicate that ProNEP has good generalization ability, which can be a valuable tool for researchers in identifying potential CNEs.

**Table 3.** Results of two plant-pathogen systems.

Experiment	True positives	False positives	False negatives	True negatives	Accuracy
*Arabidopsis thaliana—Hyaloperonospora arabidopsidis* system					
1	0	0	0	100	1
2	0	0	0	100	1
3	0	0	0	100	1
4	0	0	0	100	1
5	0	0	0	100	1
6	2	0	0	98	1
7	3	2	0	95	0.98
8	2	3	0	95	0.97
9	1	1	0	98	0.99
10	3	1	0	96	0.99
*Triticum aestivum—Blumeria graminis* system					
1	0	4	1	95	0.95
2	1	3	2	94	0.95
3	0	3	1	96	0.96
4	0	2	2	96	0.96
5	5	3	1	91	0.96
6	5	2	2	91	0.96
7	4	1	2	93	0.97
8	7	2	1	90	0.97
9	0	0	2	98	0.98
10	3	2	0	95	0.98

**Table 4.** Detailed information on nine newly collected CNEs.

NLR	Plant	Effector	Pathogen	References
*CSA1*	*Arabidopsis thaliana*	*HopB*	*Pseudomonas syringae*	Schulze *et al.* (2022)
*Mla1* (Crean et al., 2023)	*Hordeum vulgare*	*Avra_1_*	*Blumeria graminis*	Crean *et al.* (2023)
*Mla3* (Brabham et al., 2024)	Hordeum vulgare	Pwl2	Magnaporthe oryzae	Brabham *et al.* (2024)
*Mla3* (Brabham et al., 2024)	*Hordeum vulgare*	*Pwl2-2*	*Magnaporthe oryzae*	Brabham *et al.* (2024)
*Mla13* (Crean et al., 2023)	*Hordeum vulgare*	*Avra_13-1_*	*Blumeria graminis*	Crean *et al.* (2023)
*Mla13* (Crean et al., 2023)	*Hordeum vulgare*	*Avra_13-2_*	*Blumeria graminis*	Crean *et al.* (2023)
*Mla13* (Crean et al., 2023)	*Hordeum vulgare*	*Avra_13-3_*	*Blumeria graminis*	Crean *et al.* (2023)
*Roq1* (Prautsch et al., 2023)	*Nicotiana benthamiana*	*ripb*	*Ralstonia solanacearum*	Prautsch *et al.* (2023)
*Roq1* (Prautsch et al., 2023)	Nicotiana benthamiana	XopQ	Ralstonia solanacearum	Prautsch *et al.* (2023)

## 4 Discussion

NLRs play a crucial role in plant resistance against a wide range of pathogens, including fungi, bacteria, oomycetes, and viruses (Belkhadir *et al.* 2004, Collier and Moffett 2009). They can recognize pathogen effectors and activate the immune response to defend against diseases. Identifying NLRs and their corresponding effectors is crucial, as it facilitates the intraspecific (Vuong *et al.* 2023) and interspecific (Orbegozo *et al.* 2016) transfer of NLRs through genetic engineering, contributing significantly to the enhanced disease defense. In this study, we introduce ProNEP, the first deep learning tool for the precise identification of NLR-effector pairs. ProNEP offers a powerful and efficient means to identify the interactions between NLRs and effectors in a high-throughput way, thereby advancing our understanding of plant immunity and enabling targeted strategies for enhancing crop resistance.

ProNEP demonstrates superior performance by leveraging both the transfer learning and the bilinear attention mechanisms. Transfer learning based on the pre-trained models has demonstrated significant potential in addressing protein-related challenges. For example, the CLEAN (Yu *et al.* 2023), built upon a 650-million-parameter protein language model (ESM1b; Rives *et al.* 2021), stands out for its superior performance in predicting enzyme functions. In the prediction of PPI, the D-SCRIPT (Sledzieski *et al.* 2021), utilizing a structure-based pre-trained protein language, have also outperformed other methods. Simultaneously, transfer learning exhibits robust adaptability and generalization capabilities when confronted with small-sample data. In our study, transfer learning augments protein information for CNE identification, which greatly enhances model performance. On the other hand, the acquisition of interaction representation is pivotal for the identification of CNEs, which can be effectively achieved through the bilinear attention network. The bilinear attention network was originally designed to address visual question answering (VQA) problems, focusing on pairs of image regions and question words to learn their interaction representation (Kim *et al.* 2018). The bilinear attention network has achieved notable success in several protein-related issues, particularly excelling in the domain of drug target prediction (Drug-BAN; Bai *et al.* 2023). However, its application in PPI research remains limited. D-SCRIPT, for instance, only uses a residue contact module to predict interactions between proteins. Topsy-Turvy builds on D-SCRIPT, combining it with global and local integrated diffusion embedding (GLIDE). GLIDE predicts PPI by quantifying the likelihood of interactions between each pair of proteins in a network through a combination of local (proximity-based) and global (diffusion state embedding-based) graph theory techniques. Despite using the same feature engineering strategies, D-SCRIPT and Topsy-Turvy do not perform as well as ProNEP, which employs a bilinear attention mechanism that precisely focuses on critical residue pair information in protein sequences. We expect the introduction of the bilinear attention network to improve the performance of PPI models by leveraging its powerful interactive representation learning capabilities.

ProNEP has three functions: Identify effectors for given NLRs; identify NLRs for given effectors; and calculate the interaction probability for given pairs of NLRs and effectors. We used ProNEP to identify 23 310 potential CNE interactions from 91 291 NLRs in public databases (Liu *et al.* 2021), involving 111 effectors. We have uploaded the results to the NERRD database. However, we also recognize that the diversity of effectors in real-world scenarios far exceeds the range represented in our current dataset. To mitigate the potential limitation brought by the diversity of effectors in our database, we recommend using two functions of ProNEP, “identify NLRs for given effectors” and “calculate the interaction probability for given pairs of NLRs and effectors.” These functions allow researchers to use their own effectors and NLRs that are not included in the database to predict potential interactions. In further study, we plan to enrich the diversity within our database by incorporating a larger and more diverse set of effector data to enhance the study of plant immune mechanisms. In summary, we anticipate widespread applications of ProNEP in predicting CNEs for more species, thereby advancing research in plant biology (Tamborski and Krasileva 2020), plant immunology (Yuan *et al.* 2021), and crop breeding (Zhang *et al.* 2023).

## Supplementary Material

btae581_Supplementary_Data
