# Supplementary material for: Identifying nucleotide-binding leucine-rich repeat receptor and pathogen effector pairing using transfer-learning and bilinear attention network
Source: Bioinformatics. 2024 Sep 27;40(10):btae581. doi: 10.1093/bioinformatics/btae581 (PMC11969219; doi:10.1093/bioinformatics/btae581)
Supplement: btae581_Supplementary_Data [file btae581_supplementary_data.zip › Supplementary Table 5.docx]

**Supplementary Table 5.** Performance comparison of different pre-trained model on the independent test dataset.

| Model | Size | Accuracy | Precision | Sensitivity | F1-score | AUROC | AUPRC |
| --- | --- | --- | --- | --- | --- | --- | --- |
| ESM-1b | 345M | 0.7802 | 0.8 | 0.4706 | 0.5936 | 0.7664 | 0.3406 |
| ProtTrans | 10.5G | 0.91 | 0.8029 | 0.9091 | 0.8527 | 0.9060 | 0.7210 |
| ProNEP | 7.2G | 0.914 | 0.945 | 0.915 | 0.928 | 0.966 | 0.747 |
