# Supplementary material for: Identifying nucleotide-binding leucine-rich repeat receptor and pathogen effector pairing using transfer-learning and bilinear attention network
Source: Bioinformatics. 2024 Sep 27;40(10):btae581. doi: 10.1093/bioinformatics/btae581 (PMC11969219; doi:10.1093/bioinformatics/btae581)
Supplement: btae581_Supplementary_Data [file btae581_supplementary_data.zip › Supplementary Table 2.docx]

**Supplementary Table 2.** Model performance with 10 different train datasets on the independent test dataset.

| Model | Accuracy | Precision | Recall | F1-score | AUROC | AUPRC |
| --- | --- | --- | --- | --- | --- | --- |
| Train1 | 0.919 | 0.8889 | 0.9194 | 0.9039 | 0.9424 | 0.6965 |
| Train2 | 0.9075 | 0.9202 | 0.9189 | 0.9132 | 0.9605 | 0.7920 |
| Train3 | 0.9203 | 0.9444 | 0.9174 | 0.9307 | 0.9526 | 0.7233 |
| Train4 | 0.9169 | 0.906 | 0.9161 | 0.911 | 0.9617 | 0.76 |
| Train5 | 0.9231 | 0.9332 | 0.919 | 0.9261 | 0.9733 | 0.7126 |
| Train6 | 0.9075 | 1 | 0.8981 | 0.9463 | 0.9768 | 0.7582 |
| Train7 | 0.945 | 0.973 | 0.9421 | 0.9573 | 0.9736 | 0.6824 |
| Train8 | 0.915 | 0.973 | 0.9091 | 0.94 | 0.9825 | 0.8551 |
| Train9 | 0.845 | 0.973 | 0.832 | 0.897 | 0.9605 | 0.7478 |
| Train10 | 0.945 | 0.9421 | 0.973 | 0.9573 | 0.9736 | 0.6824 |
| Mean | 0.91443 | 0.94538 | 0.91451 | 0.92828 | 0.96575 | 0.74703 |
| SD | 0.027733976 | 0.03465195 | 0.03553241 | 0.0217456 | 0.01234947 | 0.05404823 |
